# Identification of nucleotide sequence involved in Weissellicin L production

**DOI:** 10.1186/2193-1801-3-617

**Published:** 2014-10-18

**Authors:** Yi-sheng Chen, Yun-shien Lee, Hui-chung Wu, Chih-ming Chiang, Shwu-fen Pan, Kun-hon Leong

**Affiliations:** Department of Biotechnology, Ming Chuan University, No. 5, De-Ming Rd., Gui-Shan Township, Taoyuan County, 333 Taiwan

**Keywords:** Weissellicin L, *Weissella hellenica*, Bacteriocin

## Abstract

**Background:**

Weissellicin L, a novel bacteriocin produced by *Weissella hellenica* 4*–*7, was previously characterized but its full amino acid sequence remain unknown. The draft genome sequencing analysis of *Weissella hellenica* 4*–*7 was performed and the open reading frame (ORF) encoding the weissellicin L was identified and clarified.

**Findings:**

The obtained results indicated that the mature bacteriocin consists of 29 amino acid residues with a molecular weight of approximately 3205.64 Da. A conserved processing site of two glycine residues in positions -1 and -2 was observed in the leader peptides. The possibility that bacteriocin secretion depended on ATP-binding cassette (ABC) transporter was therefore suggested. Furthermore, primers were designed from 5’ and 3’ flanking sequences of the weissellicin L structural gene. PCR presented a single product and was useful to detect weissellicin L structural gene.

**Conclusions:**

To our knowledge, this is the first report describing the full amino acid sequence of Weissellicin L. A rapid method to detect weissellicin L structural gene was also reported in this study.

## Introduction

It has been frequently found that parts of lactic acid bacteria (LAB) strains produce proteinaceous antibacterial compounds, termed as bacteriocins. Many bacteriocins show great inhibitory ability against food pathogens and therefore attract special interest (Klaenhammer 1988; Ennahar et al. 1999; Cleveland et al. 2001; Yang et al. 2014). In the previous studies, we reported that *Weissella hellenica* 4*–*7, isolated from the traditional Taiwanese fermented food *sian-sianzih* (fermented clams), is capable of producing a novel bacteriocin, termed weissellicin L (Leong et al. 2013).

Several characteristics of weissellicin L, such as sensitivities to enzymes and heat, inhibition spectra, and partial amino acid sequences, have been previously reported (Leong et al. 2013). Results obtained from mass spectrometry analysis revealed the bacteriocin mass of weissellicin L was approximately 3205.64 Da. However, only 17 amino acid residues from *N*-terminal have been clarified. The objectives of this study are quite simple, 1) to clarify the full amino acid sequence of weissellicin L, and 2) to rapidly detect weissellicin L structural gene by using PCR amplification method.

## Materials and methods

### DNA extraction

Strain *W. hellenica* 4–7 was grown in a modified Glucose Yeast Peptone (GYP) medium under the same conditions previously described by Leong et al. (2013). Genomic DNA was extracted from cells and purified using the Qiagen Blood & Cell Culture DNA kit (Qiagen, Hilden, Germany)(Aguado-Urda et al. 2011).

### Draft genome sequencing analysis

Illumina GA IIx genome analyzer (Illumina, San Diego, CA) was applied to reveal the genome sequence of *W. hellenica* 4–7. Using *de novo* Velvet algorithms, short reads obtained were assembled to generate a single chromosome sequence (Chiu et al. 2013). Sequence comparison was carried out using the previously obtained partial *N*-terminal sequence of weissellicin L (Leong et al. 2013) against the draft genome of *W. hellenica* 4–7 (unpublished). Search for similarity between sequences was performed using NCBI BLAST (http://blast.ncbi.nlm.nih.gov/).

### Design of primers

**Figure 1:**
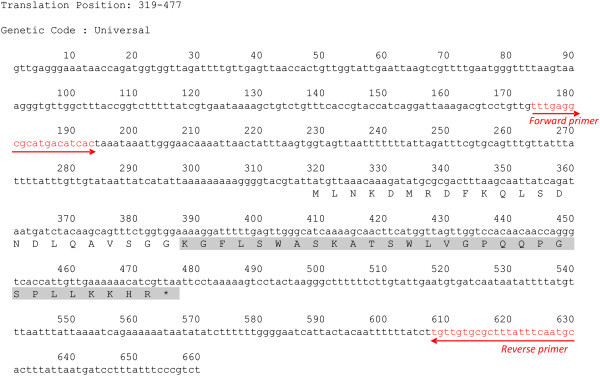
**Nucleotide sequence of the weissellicin L gene and the deduced amino acid sequence.** An asterisk indicates the translation stop site. The mature weissellicin L peptide is highlighted in grey.

Weissellicin L-specific oligonucleotide primers were designed from the 5’ and 3’ flanking sequences of the weissellicin L structural gene sequence by using NCBI/ Primer-BLAST tool (http://www.ncbi.nlm.nih.gov/tools/primer-blast/) (Figure 1). The primer sequences were as follow: 4-7bat-SF (5’-GCATTGAAATAAAGCGCACAACA-3’) and 4-7bat-SR (5’- TTTGAGGCGCATGACATCAC-3’). The oligonucleotides were synthesized by Genomics BioSci & Tech Ltd. (New Taipei City, Taiwan).

### *Weissella*strains and PCR conditions

Besides *W. hellenica* 4–7, strains *W. hellenica* BCRC 80264^T^ obtained from the Bioresource Collection and Research Center (BCRC, Hsinchu, Taiwan) and *W. hellenica* 203 previously isolated from fermented zoned cerith (Chen et al. 2013), were used as the negative controls. Neither strain BCRC 80264^T^ nor strain 203 has bacteriocin-producing ability. Culture conditions of *W. hellenica* strains BCRC 80264^T^ and 203 were the same with strain 4–7. The thermal cycling parameters were an initial denaturation at 95°C for 3 min and 30 s for subsequent cycles, primer annealing at 59°C for 30 s and primer extension at 72°C for 1 min. There were 30 cycles followed by a final extension at 72°C for 10 min. The PCR products were visualized on a 2% agarose gel in 1× TAE. A 100-bp DNA ladder marker (Genomics BioSci & Tech Ltd., New Taipei City, Taiwan) was used as the size standard.

## Results and discussion

In our previous study, a new bacteriocin termed weissellicin L was identified in *Weissella hellenica* 4*–*7 but only partial *N*-terminal amino acid sequence was observed and the complete sequence remained unknown (Leong et al. 2013). The whole genome of strain *Weissella hellenica* 4*–*7 was analyzed afterward to create a draft genome sequence (unpublished). The previously identified partial *N*-terminal amino acid sequence of weissellicin L, NH_2_-KGFLSWASKATSWLVGP, was applied to search against the draft genome of *W. hellenica* 4–7. An open reading frame was detected to match the partial sequence of weissellicin L completely.

The deduced bacteriocin comprised 52 amino acid residues in the full length precursor peptide and 29 residues in the mature peptide (Figure 1). The molecular weight of the deduced 29 amino acid residues was calculated to be 3205.76 Da using Compute pl/Mw tool in the ExPASy Proteomics Server (http://web.expasy.org/compute_pi/). This calculated molecular weight corresponded to the previously determined molecular weight of 3205.64 Da using MALDI-TOF MS (Leong et al. 2013). Therefore, the nucleotide sequences encoding the putative structural gene for weissellicin L and its flanking regions were revealed. The sequences determined in this study have been deposited in the DDBJ database with accession number AB983710.

The activity of a dedicated ATP-binding cassette (ABC) transporter is required for the secretion of many class II bacteriocin in Gram-positive bacteria (Michiels et al. 2001). A double-glycine-type leader could always be observed with two glycine residues located at positions -1 and -2 of the leader peptides. This double-glycine sequence is a hallmark of the class II bacteriocins exported through ABC transporters (Dimov et al. 2005; Michiels et al. 2001). In this study, the double-glycine-type leader sequence also presented at the same positions of the leader peptide (Figure 1). It is therefore suggested that weissellicin L was exported through ABC transporters in *W. hellenica* 4–7. However, without indepth study the molecular interaction and detailed mechanism of the secretion is not discussed here.

**Figure 2:**
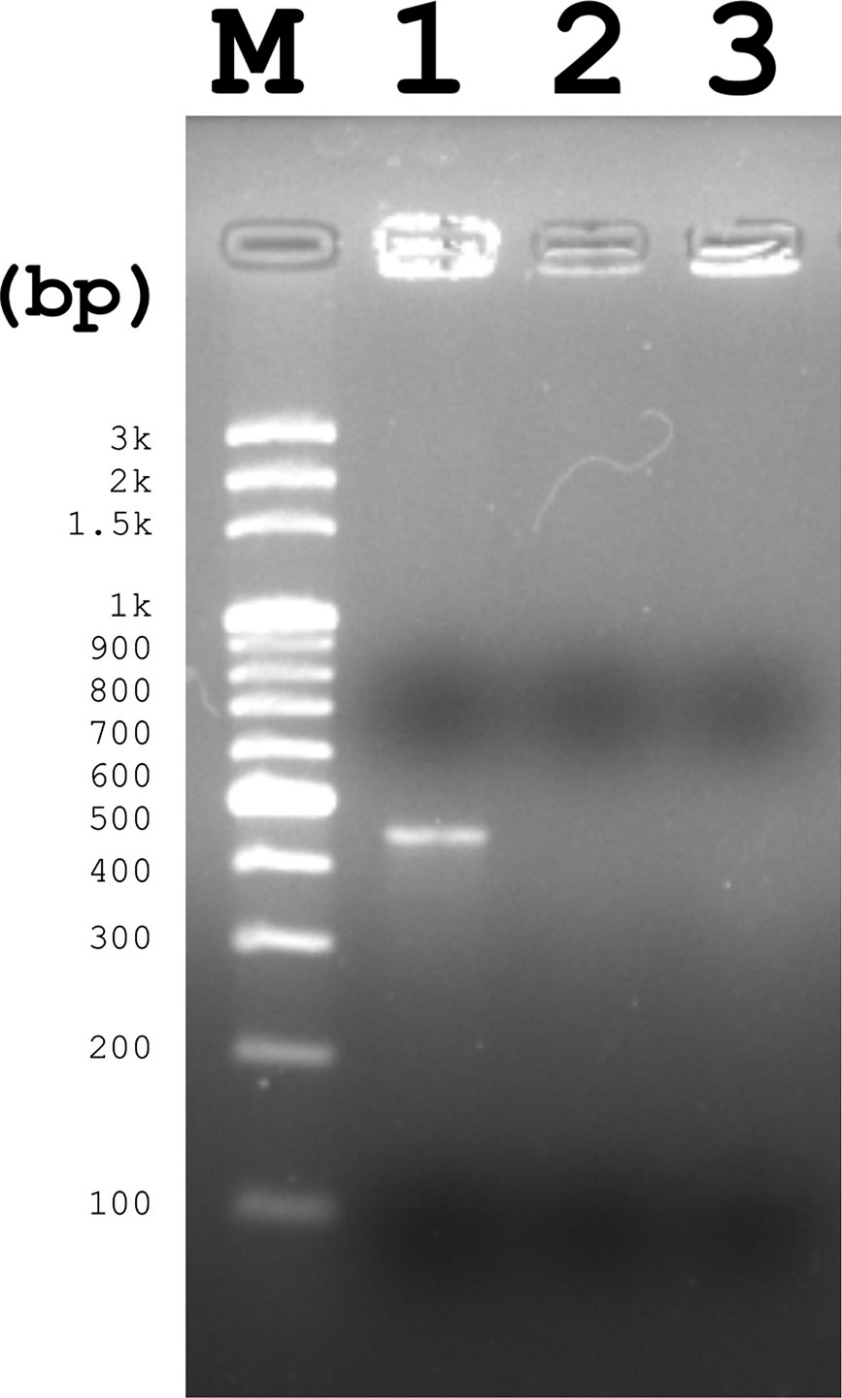
**Ethidium bromide stained 2% agarose gel of PCR products using weissellicin L gene specific primers.** Lane M, a 100-bp DNA ladder marker; Lane 1, *W. hellenica* 4–7; Lane 2, *W. hellenica* BCRC 80264^T^; Lane 3, *W. hellenica* 203.

Primers specific for the weissellicin L gene were designed to perform PCR amplification. A single 457-bp fragment was amplified from the genomic DNA of *W. hellenica* 4–7 (Figure 2). However, no amplified PCR product was observed from *W. hellenica* strains BCRC 80264^T^ and 203 (Figure 2). PCR product from *W. hellenica* 4–7 was sequenced and the nucleotide sequences encoding the target gene was confirmed to be identical as reported (Figure 1). The results suggested that PCR amplification with the weissellicin L-specific primers could be useful to select potential weissellicin L-producing strain from multitudinous *W. hellenica* strains.

Our results report the full amino acid sequences of weissellicin L and the nucleotide sequences encoding the weissellicin L gene. In addition, this study provides a quick method to screening the weissellicin L-producing strain. Further analyses on the genome sequences of *W. hellenica* 4–7 are necessary to understand more bacteriocin related information and other characteristics of LAB.
